# Prognostic value of microRNAs in gastric cancer: a meta-analysis

**DOI:** 10.18632/oncotarget.18590

**Published:** 2017-06-21

**Authors:** Yue Zhang, Dong-Hui Guan, Rong-Xiu Bi, Jin Xie, Chuan-Hua Yang, Yue-Hua Jiang

**Affiliations:** ^1^ First Clinical Medical College, Shandong University of Traditional Chinese Medicine, Jinan 250355, Shandong, People's Republic of China; ^2^ Department of Orthopedics, Affiliated Hospital of Shandong University of Traditional Chinese Medicine, Jinan 250011, Shandong, People's Republic of China; ^3^ Department of Cardiology, Affiliated Hospital of Shandong University of Traditional Chinese Medicine, Jinan 250011, Shandong, People's Republic of China; ^4^ Central Laboratory, Affiliated Hospital of Shandong University of Traditional Chinese Medicine, Jinan 250011, Shandong, People's Republic of China

**Keywords:** microRNA, gastric cancer, prognosis, meta-analysis

## Abstract

**Background:**

Previous articles have reported that expression levels of microRNAs (miRNAs) are associated with survival time of patients with gastric cancer (GC). A systematic review and meta-analysis was performed to study the outcome of it.

**Design:**

Meta-analysis.

**Methods:**

English studies estimating expression levels of miRNAs with any of survival curves in GC were identified up till March 19, 2017 through performing online searches in PubMed, EMBASE, Web of Science and Cochrane Database of Systematic Reviews by two authors independently. The pooled hazard ratios (HR) with 95% confidence intervals (CI) were used to estimate the correlation between miRNA expression and overall survival (OS).

**Results:**

Sixty-nine relevant articles about 26 miRNAs with 6148 patients were ultimately included. GC patients with high expression of miR-20b (HR=2.38, 95%CI=1.16-4.87), 21 (HR=1.77, 95%CI=1.01-3.08), 106b (HR=1.84, 95%CI=1.15-2.94), 196a (HR=2.66, 95%CI=1.94-3.63), 196b (HR=1.67, 95%CI=1.38-2.02), 214 (HR=1.84, 95%CI=1.27-2.67) or low expression of miR-125a (HR=2.06, 95%CI=1.26-3.37), 137 (HR=3.21, 95%CI=1.68-6.13), 141 (HR=2.47, 95%CI=1.34-4.56), 145 (HR=1.62, 95%CI=1.07-2.46), 146a (HR=2.60, 95%CI=1.63-4.13), 206 (HR=2.85, 95%CI=1.73-4.70), 218 (HR=2.61, 95%CI=1.74-3.92), 451 (HR=1.73, 95%CI=1.19-2.52), 486-5p (HR=2.45, 95%CI=1.65-3.65), 506 (HR=2.07, 95%CI=1.33-3.23) have significantly poor OS (P<0.05).

**Conclusions:**

In summary, miR-20b, 21, 106b, 125a, 137, 141, 145, 146a, 196a, 196b, 206, 214, 218, 451, 486-5p and 506 demonstrate significantly prognostic value. Among them, miR-20b, 125a, 137, 141, 146a, 196a, 206, 218, 486-5p and 506 are strong biomarkers of prognosis in GC.

## INTRODUCTION

Great quantities of previous articles have reported that expression levels of microRNAs (miRNAs) are associated with survival time of gastric cancer (GC) patients [1–167]. GC is still the fourth most common cancer all over the world and the second most universal cause of cancer death globally, although there has been a constant descent in morbidity and mortality in the past few decades [168, 169]. The early clinical inspection of GC was under 15%, and cases of advanced GC accounted for 85% [170]. At present, the primary treatment choices are surgical intervention, chemotherapy, immunogene therapy, and target therapy. The clinical result of GC mainly depends on the stage of tumor. Unfortunately, GC patients’ median survival time is no more than 6-9 months [171]. It is unlimited proliferation of cancer cells and ability of intense invasive and metastasis that mainly causes high malignancy degree and poorer survival time. As a result, a novel diagnostic means and improved prognosis of GC might be created through identification of molecular aberrations, which can predict cancer progression and survival rate.

During the past decade, the associations between non-coding RNAs (ncRNAs) and GC have been widely researched. Generally speaking, ncRNAs have been classified as small ncRNAs, consisting of miRNAs and long non-coding RNAs (lncRNAs).

MiRNAs, a novel class of small (20-24 nucleotides [nt]) non-coding regulatory RNAs, play a significant role in multiple biological processes, such as cell division, differentiation, senescence and apoptosis [172, 173]. An increasing number of evidence shows that various miRNAs are unconventionally expressed in diverse types of human cancers, and a few miRNAs have been shown to be related with tumor formation, development, progression, and response to treatment by miRNA expression profiling [174].

Moreover, a series of studies have already demonstrated that lncRNAs also play crucial roles in GC progression. A previous investigation reported that, compared with non-tumor tissues, H19 was one of the most elevated lncRNAs with a ˜8.91-fold change in human primary GC [175]. In addition, Li et al. [176] recognized certain potential lncRNAs that abnormally expressed between GC and normal tissues by screening a cohort of 74 GC patients as well, among which, H19 was chosen as a result of a significant overexpression. Furthermore, expression levels of the lncRNAs H19, ANRIL, GHET1, HOTAIR, GAS5, LET, GAPLINC and FENDRR are also significantly associated with the 5-year survival rate of GC patients [176–183].

In GC research area, quite a number of investigations have demonstrated that miRNAs are associated with survival time of patients [1–167]. However, the number of patients during the articles mentioned above is generally not big enough. Therefore, a systematic review and meta-analysis was performed for the sake of better understanding accurate prognostic value between expression levels of numerous miRNAs and HR of GC patients.

## RESULTS

### Study selection

**Figure 1:**
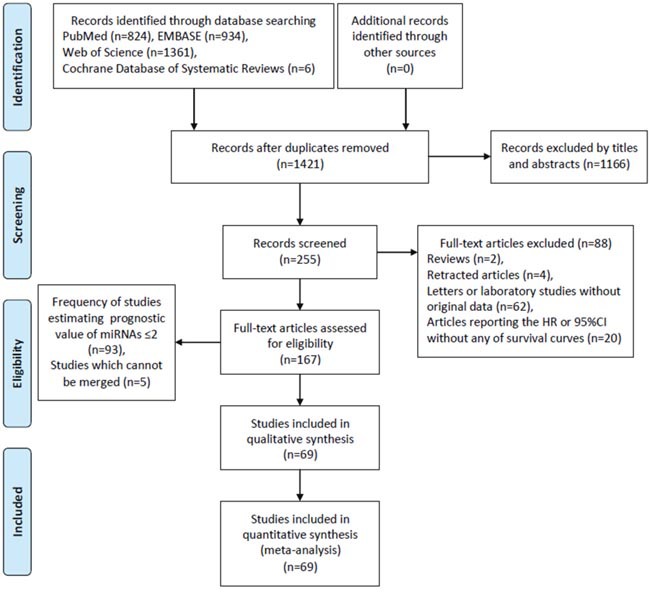
Flow diagram of literature search and selection

A flow diagram with details of the study selection process was presented in Figure 1.

### Study frequency

Frequency of studies estimating prognostic value of miRNAs in GC were shown in Table 1 (highlighted studies were included in the present meta-analysis), including miRNA name, number of studies estimating prognostic value, and reference.

**Table 1:** Frequency of studies estimating prognostic value of miRNAs in gastric cancer

miRNA	N	R	miRNA	N	R	miRNA	N	R	miRNA	N	R	miRNA	N	R	miRNA	N	R	miRNA	N	R
let-7g	1	1	27b	3	32-34	126	2	54,55	148a	1	78	200a	1	3	328	1	126	485-5p	1	146
10b	1	2	29a	2	35,36	128	2	32,34	150-5p	1	77	200b	2	96,97	335	3	41,127,128	486-5p	3	56,147,148
15a	1	3	29b	1	36	129-5p	1	56	150	2	12,79	200c	4	66,96,98,99	337-3p	1	129	493	1	149
16	2	3,4	29c	1	36	130a	1	57	153	1	80	203	2	100,101	340	1	130	494	1	150
17-5p	2	5,6	29	1	19	132	1	58	181a-5p	1	81	204	2	102,103	342-3p	1	79	500	1	151
18a	2	7,8	31	1	37	133a-3p	1	56	181b	1	16	206	3	104-106	361-5p	1	131	501-5p	1	152
19a	1	9	34a	5	3,38-41	133	2	59,60	181c	1	82	210	1	107	363	1	132	503	1	153
19b	1	10	92a	2	11,42	135a	1	61	182-5p	1	56	211	1	108	375	1	68	506	3	154-156
20a	3	3,5,11	93	2	43,44	135b-5p	1	56	183-5p	1	56	212	1	109	377	1	133	508-5p	2	33,157
20b	3	3,12,13	100	1	34	135b	1	61	183	2	83,84	214	4	1,34,110,111	378	1	134	520c	1	158
21-5p	1	14	101	2	32,34	137	3	62-64	185	2	3,85	215	1	87	381	1	135	520d-3p	1	159
21	7	3,6,15-19	103	1	3	141	3	65-67	187	1	86	217	2	112,113	421	2	136,137	558	1	160
22	2	20,21	106a	2	3,6	142-5p	1	68	192	3	48,79,87	218	3	114-116	425	1	3	590-5p	1	161
23b-3p	1	22	106b	3	3,6,45	143	3	3,69,70	193b	1	88	221	2	117,118	429	1	138	630	1	162
23b	1	23	107	3	3,46,47	144-5p	1	56	194	1	89	222	2	118,119	433	1	1	873	1	163
24	1	24	122	1	48	144	1	71	196a	4	88,90-92	223	2	120,121	448	1	139	939	1	164
25	2	25,26	125a-3p	1	49	145-5p	2	56,72	196b-5p	1	56	224	2	79,122	449c	1	140	940	1	165
26a	2	27,28	125a-5p	1	50	145	2	34,73	196b	3	91-93	300	1	123	451	4	4,141-143	1207-5p	1	166
26b	1	29	125a	1	51	146a	3	74-76	198	1	94	301a	1	124	452	1	144	1225-5p	1	167
27a	2	30,31	125b	2	52,53	146b-5p	1	77	199a	1	95	326	1	125	455-5p	1	145	1266	1	166

Highlighted studies were included in the present meta-analysis; N: Number of studies estimating prognostic value; R: Reference.

### Study characteristics

Characteristics of articles with Kaplan-Meier survival curves in GC were comprehensively detailed in Table 2, including miRNA name, names of the first authors, publication year, reference number, country, study design, detected sample, number of patients, stage, cut-off value, main miRNA method, maximum months of follow-up, survival analysis and HR of low or high expression on the basis of relevant survival analysis with 95%CI. If the data were not provided visually and only as Kaplan-Meier survival curves, the data were extracted from the graphical survival plots, and estimations of the HR with 95%CI were then performed using a previously described method [184] with the software Engauge Digitizer version 4.1. Furthermore, if both the univariate and multivariate results were reported, then only the latter was selected, since these results were adjusted for confounding factors.

**Table 2:** Characteristics of articles with Kaplan-Meier survival curves in gastric cancer

miRNA	Study	Country	Study design	Sample	Number	Stage	Cut-off	Method	Follow-up (month)	Result	HR(L/H)	HR(H/L)	95%CI
20a	Osawa S, 2011 [3]	Japan	R	FFPE	37	II-III	70%	qRT-PCR	60	OS^u^		1.93	0.48-7.87
20a	Wang M, 2012 [5]	China	R	Plasma	65	I-IV	0.26	RT-qPCR	36	OS^m^		1.58	1.10-2.25
20a	Wu Q, 2013 [11]	China	R	FFPE	97	None	Median	qRT-PCR	66	OS^m^		1.01	1.00-1.02
20b	Katada T, 2009 [12]	Japan	R	Frozen	42	None	None	qRT-PCR	60	OS^m^		2.01	0.59-6.85
20b	Osawa S, 2011 [3]	Japan	R	FFPE	34	II-III	70%	qRT-PCR	60	OS^u^		1.21	0.20-7.23
20b	Xue TM, 2015 [13]	China	R	Tissue	102	I-IV	Median	RT-qPCR	75	OS^m^		3.32	1.20-9.14
21-5p	Park SK, 2016 [14]	Korea	R	FFPE	50	III	ROC	qRT-PCR	168	RFS^u^		2.05	1.26-3.34
21	Jiang J, 2011 [15]	China	R	FFPE	55	III-IV	None	qRT-PCR	17	OS^u^		5.88	2.22-16.67
21	Osawa S, 2011 [3]	Japan	R	FFPE	33	II-III	70%	qRT-PCR	60	OS^u^		2.58	0.34-19.79
21	Xu Y, 2012 [16]	China	R	Frozen	86	I-IV	5.12	qRT-PCR	36	OS^u^		1.15	0.59-2.25
21	Hirata K, 2013 [17]	Japan	P	Tissue	61	None	3.58	IHC	42	RFS^u^		0.82	0.27-2.43
21	Komatsu S, 2013 [6]	Japan	R	Plasma	69	I-IV	0.03	qRT-PCR	40	CSS^m^		13.39	1.72-104.42
21	Song J, 2013 [18]	China	R	Serum	103	I-IV	0.64	qRT-PCR	54	OS^u^		0.99	0.48-2.07
21	Wang D, 2015 [19]	China	R	Tissue	50	I-IV	ROC	qRT-PCR	12	OS^u^		1.89	1.17-3.07
27b	Liu HT, 2015 [32]	China	R	FFPE	103	I-IV	None	qRT-PCR	66	OS^u^	0.80		0.46-1.41
27b	Shang Y, 2016 [33]	China	R	Tissue	114	I-IV	None	ISH	84	OS^u^	1.61		0.92-2.80
27b	Liu HT, 2017 [34]	China	R	FFPE	102	I-IV	Median	RT-qPCR	67	OS^m^	1.33		0.60-2.98
34a	Osawa S, 2011 [3]	Japan	R	FFPE	37	II-III	70%	qRT-PCR	60	OS^u^	0.20		0.06-0.68
34a	Hui WT, 2015 [38]	China	R	Frozen	76	I-III	Mean	qRT-PCR	>60	OS^m^	2.33		1.10-4.93
34a	Wei B, 2015 [39]	TCGA	R	Tissue	157	I-IV	X-tile	Downloaded	>100	OS^u^	2.31		0.13-40.12
34a	Zhang H, 2015 [40]	China	R	Frozen	137	I-IV	2.44	qRT-PCR	68	OS^m^	1.33		1.14-1.61
34a	Yang B, 2016 [41]	China	R	Tissue	50	I-IV	Median	qRT-PCR	60	OS^u^	3.05		0.60-15.50
106b	Osawa S, 2011 [3]	Japan	R	FFPE	37	II-III	70%	qRT-PCR	60	OS^u^		2.70	0.43-17.06
106b	Komatsu S, 2013 [6]	Japan	R	Plasma	69	I-IV	0.05	qRT-PCR	40	CSS^u^		1.22	0.52-2.84
106b	Yang TS, 2014 [45]	China	R	Tissue	120	None	Median	qRT-PCR	45	OS^u^		1.79	1.10-2.90
107	Li X, 2011 [46]	China	R	FFPE	50	None	90.95	qRT-PCR	48	OS^u^		0.48	0.28-0.82
107	Osawa S, 2011 [3]	Japan	R	FFPE	37	II-III	70%	qRT-PCR	60	OS^u^		4.09	1.26-13.32
107	Inoue T, 2012 [47]	Japan	R	Frozen	161	I-IV	2.74	RT-qPCR	60	OS^m^		2.21	1.18-4.61
125a-3p	Hashiguchi Y, 2012 [49]	Japan	R	Frozen	70	I-IV	7.42	RT-qPCR	147.6	OS^u^	3.01		1.26-7.20
125a-5p	Nishida N, 2011 [50]	Japan	R	Frozen	87	I-IV	None	RT-qPCR	147.6	OS^u^	2.16		0.96-4.86
125a	Dai J, 2015 [51]	China	R	FFPE	73	I-IV	None	qRT-PCR	62	OS^u^	1.31		0.54-3.18
137	Gu Q, 2015 [62]	China Set IChina Set II	R	Frozen	6787	I-III	Median	qRT-PCR	96	OS^m^ OS^m^	6.802.41		2.06-22.481.13-5.11
137	Zheng X, 2015 [63]	China	R	FFPE	38	I-IV	Median	qRT-PCR	56	DFS^u^	2.70		1.18-6.17
137	Du Y, 2016 [64]	China	R	Tissue	14	I-IV	0.01	qRT-PCR	96	OS^u^	2.49		0.32-19.59
141	Lu YB, 2015 [65]	China	R	Frozen	95	I-IV	Median	qRT-PCR	60	OS^m^	2.97		1.30-10.00
141	Zhou X, 2015 [66]	China	R	Frozen	63	IIB-IV	Median	qRT-PCR	>30	DFS^u^	2.47		1.22-5.00
141	Huang M, 2016 [67]	China	R	Frozen	30	I-IV	None	qRT-PCR	26.83	OS^u^	2.23		1.04-4.79
143	Osawa S, 2011 [3]	Japan	R	FFPE	37	II-III	70%	qRT-PCR	60	OS^u^		2.95	0.19-46.23
143	Naito Y, 2014 [69]	Japan	R	Frozen	66	I-IV	1/3	qRT-PCR	50	CSS^m^		2.62	1.21-5.80
143	Li JH, 2016 [70]	China	R	Frozen	44	I-IV	1.18	qRT-PCR	26	OS^u^		0.40	0.23-0.70
145-5p	Zhang Y, 2016 [72]	China	R	Frozen	145	I-IV	None	RT-qPCR	66	OS^m^	3.87		1.13-11.44
145-5p	Li CY, 2017 [56]	TCGA	R	Tissue	361	I-IV	None	Downloaded	60	OS^u^	1.37		1.08-1.74
145	Naito Y, 2014 [73]	Japan	R	FFPE	71	I-IV	Median	qRT-PCR	66.67	CSS^m^	0.71		0.33-1.49
145	Liu HT, 2017 [34]	China	R	FFPE	102	I-IV	Median	RT-qPCR	67	OS^u^	1.68		0.87-3.25
146a	Kogo R, 2011 [74]	Japan	R	Frozen	90	I-IV	Median	qRT-PCR	132	OS^u^	2.20		1.31-3.70
146a	Hou Z, 2012 [75]	China	R	FFPE	30	I-IV	0.34	qRT-PCR	36	OS^u^	2.59		1.24-5.39
146a	Luo Z, 2017 [76]	China	R	Frozen	93	III-IV	ROC	RT-qPCR	72	OS^u^	7.75		1.66-35.71
150-5p	Yoon SO, 2016 [77]	Korea	R	FFPE	140118	I-IV	2.00	RT-qPCR	101.8	OS^m^ RFS^u^		0.881.84	0.37-2.090.98-3.43
150	Katada T, 2009 [12]	Japan	R	Frozen	42	None	None	qRT-PCR	60	OS^m^		6.10	0.76-50.00
150	Smid D, 2016 [79]	Czech	R	FFPE	4140	None	6.006.70	qRT-PCR	>100	OS^u^ PFS^u^		1.912.08	1.14-3.211.11-3.91
183-5p	Li CY, 2017 [56]	TCGA	R	Tissue	361	I-IV	None	Downloaded	60	OS^u^	0.64		0.47-0.87
183	Cao LL, 2014 [83]	China	R	Frozen	52	I-IV	3.55	qRT-PCR	60	OS^u^	2.83		1.31-6.10
183	Xu L, 2014 [84]	China	R	Tissue	65	I-IV	Median	RT-qPCR	102	OS^u^	1.94		1.11-3.39
192	Chen Q, 2014 [48]	China	R	Plasma	61	III-IV	2.00	qRT-PCR	43	OS^m^		0.89	0.39-2.04
192	Xu YJ, 2015 [87]	China	R	Frozen	38	I-IV	None	qRT-PCR	81	OS^u^		0.99	0.96-1.02
192	Smid D, 2016 [79]	Czech	R	FFPE	41	None	2.30	qRT-PCR	>100	OS^u^		7.43	2.71-20.41
196a	Sun M, 2012 [90]	China	R	Frozen	31	II-IV	40.90	RT-qPCR	36	OS^u^		4.19	1.78-9.83
196a	Mu YP, 2014 [88]	China	R	Frozen	48	I-IV	5.69	qRT-PCR	60	OS^u^		2.88	1.43-5.79
196a	Tsai MM, 2014 [91]	China	R	Tissue	109	I-IV	77.30	qRT-PCR	60	OS^u^		2.27	1.50-3.43
196a	Tsai MM, 2016 [92]	China	R	Plasma	98	I-IV	1.15	qRT-PCR	72	OS^m^		3.06	1.10-8.50
196b-5p	Li CY, 2017 [56]	TCGA	R	Tissue	361	I-IV	None	Downloaded	60	OS^u^		2.07	1.37-3.13
196b	Lim JY, 2013 [93]	South Korea	R	Frozen	57	I-IV	None	qRT-PCR	75	OS^u^		1.50	1.06-2.12
196b	Tsai MM, 2014 [91]	China	R	Tissue	109	I-IV	21.70	qRT-PCR	60	OS^u^		1.55	1.16-2.06
196b	Tsai MM, 2016 [92]	China	R	Plasma	98	I-IV	0.93	qRT-PCR	72	OS^m^		2.91	1.04-8.17
200c	Valladares-Ayerbes M, 2012 [98]	Spain	R	Blood	52	I-IV	62.4	qRT-PCR	54	OS^m^ PFS^m^	0.450.44		0.22-0.920.21-0.92
200c	Tang H, 2013 [96]	China	R	Tissue	126	I-IV	2.00	qRT-PCR	58	OS^u^ DFS^u^	2.291.83		1.38-3.811.15-2.92
200c	Zhang HP, 2015 [99]	China	R	Serum	98	I-IV	Median	qRT-PCR	60	OS^m^	0.25		0.10-0.37
200c	Zhou X, 2015 [66]	China	R	Frozen	63	IIB-IV	Median	qRT-PCR	>30	DFS^u^	1.70		1.21-2.38
206	Yang Q, 2013 [104]	China	R	Tissue	98	I-IV	2.40	RT-qPCR	139	OS^m^	2.56		1.13-5.82
206	Shi H, 2015 [105]	China	R	Frozen	220	I-IV	Median	qRT-PCR	60	OS^m^	6.82		1.51-21.29
206	Hou CG, 2016 [106]	China	R	Serum	150	I-III	Median	RT-qPCR	60	OS^m^	2.39		1.16-4.91
214	Ueda T, 2010 [1]	Japan	R	Frozen	101	I-IV	None	qRT-PCR	102.33	OS^m^		2.70	1.30-5.61
214	Yang TS, 2013 [110]	China	R	Frozen	120	I-IV	None	qRT-PCR	45	OS^u^		1.77	1.06-2.96
214	Wang YW, 2014 [111]	China	R	FFPE	80	I-IV	Median	RT-qPCR	72	OS^u^		1.20	0.67-2.15
214	Liu HT, 2017 [34]	China	R	FFPE	102	I-IV	Median	RT-qPCR	67	OS^m^		2.75	1.12-6.76
218	Tie J, 2010 [114]	China	R	Frozen	40	I-IV	13.81	qRT-PCR	72	OS^u^	2.33		1.40-3.89
218	Xin SY, 2014 [115]	China	R	Serum	68	I-IV	None	qRT-PCR	36	OS^m^	3.16		1.06-9.40
218	Wang XX, 2016 [116]	China	R	Tissue	112	I-IV	Median	qRT-PCR	60	OS^m^	3.19		1.55-8.37
335	Yan Z, 2012 [127]	China	R	Both	74	I-IV	None	RT-qPCR	108	OS^u^	0.14		0.04-0.49
335	Yang B, 2016 [41]	China	R	Tissue	50	I-IV	Median	qRT-PCR	60	OS^u^	4.88		1.90-12.55
335	Zhang JK, 2017 [128]	China	R	Frozen	221	I-IV	Median	qRT-PCR	60	DFS^u^	1.65		1.11-2.45
451	Ren C, 2016 [4]	China	R	FFPE	180	I-IV	None	ISH	97.2	OS^m^	2.01		1.36-2.96
451	Bandres E, 2009 [141]	Spain	R	FFPE	45	I-III	Median	qRT-PCR	172	OS^u^ DFS^m^	2.023.70		0.76-5.381.57-8.70
451	Brenner B, 2011 [142]	Israel	R	FFPE	45	I-III	Median	qRT-PCR	50	RFS^u^	0.05		0.01-0.29
451	Su Z, 2015 [143]	China	R	FFPE	107	I-IV	Mean	qRT-PCR	72	OS^u^	1.08		0.53-2.19
486-5p	Li CY, 2017 [56]	TCGA	R	Tissue	361	I-IV	None	Downloaded	60	OS^u^	1.85		1.22-2.81
486-5p	Chen H, 2015 [147]	China	R	FFPE	84	I-IV	None	ISH	75	OS^m^	3.61		1.99-6.54
486-5p	Ren C, 2016 [148]	China	R	FFPE	84	I-IV	None	ISH	93.6	OS^m^	2.55		1.39-4.69
506	Deng J, 2015 [154]	China	R	Frozen	63	None	None	qRT-PCR	>60	OS^u^	3.05		1.19-7.79
506	Li Z, 2015 [155]	China	R	Frozen	84	I-IV	Mean	qRT-PCR	>60	OS^u^	1.76		0.73-4.27
506	Sakimura S, 2015 [156]	Japan	R	Tissue	141	I-IV	Median	qRT-PCR	>140	OS^m^	1.90		1.05-3.59

HR (L/H): hazard ratios of low expression versus high expression of miRNAs; HR (H/L): hazard ratios of high expression versus low expression of miRNAs; CI: confidence intervals; TCGA: The Cancer Genome Atlas; R: retrospective; P: prospective; FFPE: formalin-fixed paraffin-embedded; ROC: receiver operating characteristic; qRT-PCR: quantitative real-time polymerase chain reaction; RT-qPCR: reverse transcription quantitative real-time polymerase chain reaction; IHC: immunohistochemistry; ISH: *in-situ* hybridization; OS: overall survival; RFS: recurrence-free survival; CSS: cause-specific survival; DFS: disease-free survival; PFS: progression-free survival; ^u^Univariate analysis; ^m^Multivariate analysis. In order to facilitate read and statistics, studies estimating prognostic value of different miRNAs are shown in blue and white; studies which cannot be merged are shown in yellow.

### Meta-analysis

A summary of the HR evaluated from the whole combined analysis for all the miRNAs was shown in Table 3.

**Table 3:** Summary of the HR for miRNA expression in gastric cancer

miRNA	Survival analysis	Number of articles	Included references	HR	95%CI	Figure	P value	Heterogeneity (Higgins I2 statistic)	Total patients
High miR-20a	OS	3	3,5,11	1.25	0.84-1.87	3	0.27	I2=70.7%, P=0.03	199
High miR-20b	OS	3	3,12,13	2.38	1.16-4.87	3	0.02	I2=0.0%, P=0.60	178
High miR-21	RFS/CSS	3	6,14,17	2.10	0.72-6.12	2A	0.17	I2=65.6%, P=0.06	180
High miR-21	OS	5	3,15,16,18,19	1.77	1.01-3.08	2A	<0.05	I2=57.8%, P=0.05	327
Low miR-27b	OS	3	32-34	1.18	0.75-1.85	3	0.47	I2=36.1%, P=0.21	319
Low miR-34a	OS	5	3,38-41	1.25	0.59-2.65	2D	0.56	I2=68.4%, P=0.13	457
Low miR-34a	OS^m^	2	38,40	1.56	0.95-2.55	2D	0.08	I2=51.0%, P=0.15	213
High miR-106b	OS	2	3,45	1.84	1.15-2.94	3	0.01	I2=0.0%, P=0.67	157
High miR-107	OS	3	3,46,47	1.52	0.42-5.57	3	0.52	I2=88.8%, P<0.01	248
Low miR-125a	OS	3	49-51	2.06	1.26-3.37	4	<0.01	I2=0.0%, P=0.42	230
Low miR-137	OS	2	62,64	3.21	1.68-6.13	4	<0.01	I2=6.0%, P=0.35	168
Low miR-141	OS	2	65,67	2.47	1.34-4.56	4	<0.01	I2=0.0%, P=0.66	125
High miR-143	OS	2	3,70	0.68	0.12-3.81	4	0.66	I2=48.8%, P=0.16	81
Low miR-145	OS	3	34,56,72	1.62	1.07-2.46	4	0.02	I2=36.9%, P=0.21	608
Low miR-146a	OS	3	74-76	2.60	1.63-4.13	5	<0.01	I2=14.1%, P=0.31	213
High miR-150	OS	3	12,77,79	1.63	0.77-3.45	5	0.20	I2=47.8%, P=0.15	223
High miR-150	RFS/PFS	2	77,79	1.96	1.25-3.05	5	<0.01	I2=0.0%, P=0.79	158
Low miR-183	OS	3	56,83,84	1.46	0.55-3.83	5	0.45	I2=90.2%, P<0.01	478
High miR-192	OS	3	48,79,87	1.71	0.60-4.85	5	0.31	I2=87.0%, P<0.01	140
High miR-196a	OS	4	88,90-92	2.66	1.94-3.63	6	<0.01	I2=0.0%, P=0.62	286
High miR-196b	OS	4	56,91-93	1.67	1.38-2.02	6	<0.01	I2=0.0%, P=0.62	625
Low miR-200c	OS	3	96,98,99	0.65	0.16-2.64	6	0.54	I2=93.6%, P<0.01	276
Low miR-200c	PFS/DFS	3	66,96,98	1.20	0.60-2.38	6	0.61	I2=83.1%, P<0.01	241
Low miR-206	OS	3	104-106	2.85	1.73-4.70	7	<0.01	I2=0.0%, P=0.37	468
High miR-214	OS	4	1,34,110,111	1.84	1.27-2.67	7	<0.01	I2=23.0%, P=0.27	403
Low miR-218	OS	3	114-116	2.61	1.74-3.92	7	<0.01	I2=0.0%, P=0.77	220
Low miR-335	OS	2	41,127	0.85	0.03-27.50	7	0.93	I2=94.9%, P<0.01	124
Low miR-451	OS	3	4,141,143	1.73	1.19-2.52	8	<0.01	I2=14.7%, P=0.31	332
Low miR-451	DFS/RFS	2	141,142	0.46	0.01-31.06	8	0.72	I2=95.0%, P<0.01	90
Low miR-486-5p	OS	3	56,147,148	2.45	1.65-3.65	8	<0.01	I2=40.0%, P=0.19	529
Low miR-506	OS	3	154-156	2.07	1.33-3.23	8	<0.01	I2=0.0%, P=0.65	288

HR: hazard ratios; CI: confidence intervals; OS: overall survival; RFS: recurrence-free survival; CSS: cause-specific survival; PFS: progression-free survival; DFS: disease-free survival; ^m^Multivariate analysis.

### High expression of miR-21 predicts poor OS

**Figure 2:**
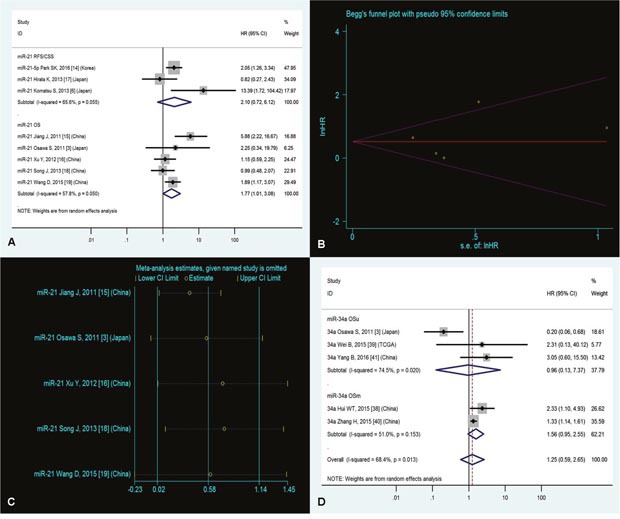
**(A)** Forest plot of the analyses about high expression of miR-21 and RFS/CSS or OS; **(B)** Publication bias of the analysis about high expression of miR-21 and OS; **(C)** Sensitivity analysis of the study about high expression of miR-21 and OS; and **(D)** Forest plot of the analyses about low expression of miR-34a and OS or OS (multivariate analysis).

Five studies [3, 15, 16, 18, 19] analyzed associations between high expression of miR-21 and OS, indicating that GC patients with high miR-21 expression had a significantly shorter OS than those with low miR-21 expression (HR=1.77, 95%CI=1.01-3.08, P<0.05, Figure 2A).

### No significant association between high expression of miR-21 and RFS/CSS

Three researches [6, 14, 17] focused on connections between high expression of miR-21 and RFS/CSS, suggesting that there was no significant association between high expression of miR-21 and RFS/CSS (HR=2.10, 95%CI=0.72-6.12, P=0.17, Figure 2A).

### Publication bias

In order to evaluate publication bias for OS of GC patients with high miR-21 expression, the Begg's funnel plot was used by us (Figure 2B). And the P value was 0.62, indicating absence of publication bias.

### Sensitivity analysis

During the study about OS of GC patients with high miR-21 expression, our sensitivity analysis did not indicate alterations in the results according to the exclusion of any individual study (Figure 2C), suggesting that no single research significantly influenced the pooled HR and the 95%CI.

### No significant association between low expression of miR-34a and OS or OS (multivariate analysis)

There was no significant association between low expression of miR-34a and OS (HR=1.25, 95%CI=0.59-2.65, P=0.56, Figure 2D) or OS (multivariate analysis, HR=1.56, 95%CI=0.95-2.55, P=0.08, Figure 2D).

### GC patients with high expression of miR-20b, 106b, 196a, 196b, 214 or low expression of miR-125a, 137, 141, 145, 146a, 206, 218, 451, 486-5p, 506 have a significantly poor OS

**Figure 3:**
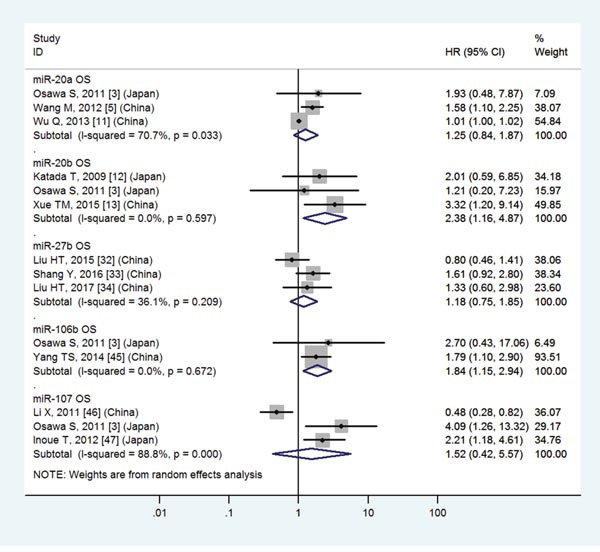
Forest plot of the analyses about high expression of miR-20a, 20b, 106b, 107 or low expression of miR-27b and OS

**Figure 4:**
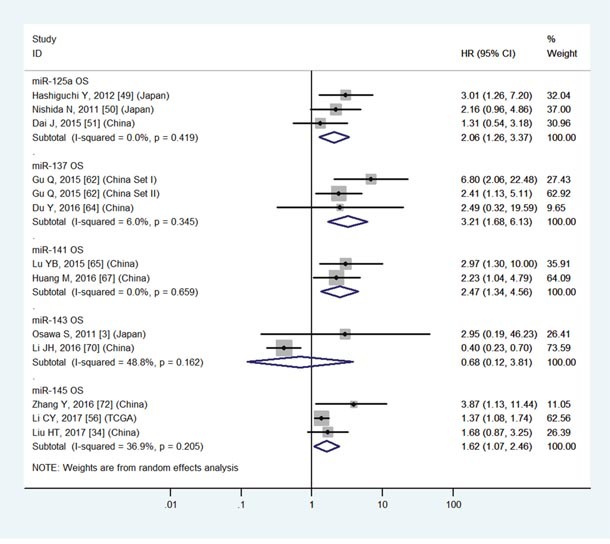
Forest plot of the analyses about high expression of miR-143 or low expression of miR-125a, 137, 141, 145 and OS

**Figure 5:**
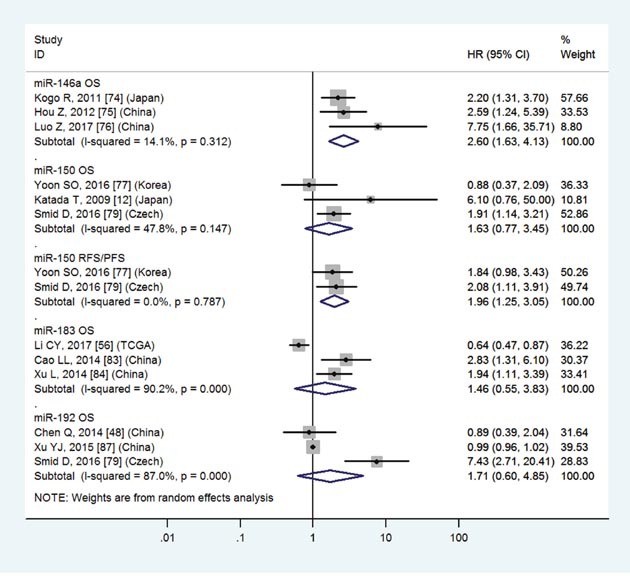
Forest plot of the analyses about high expression of miR-150, 192 or low expression of miR-146a, 183 and OS or RFS/PFS

**Figure 6:**
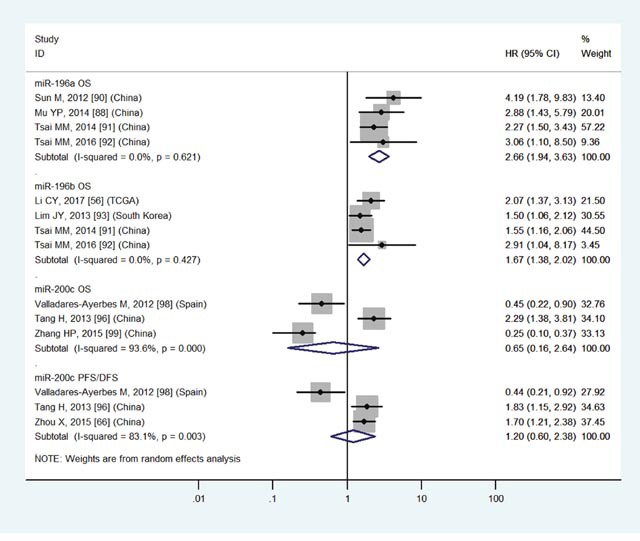
Forest plot of the analyses about high expression of miR-196a, 196b or low expression of miR-200c and OS or PFS/DFS

**Figure 7:**
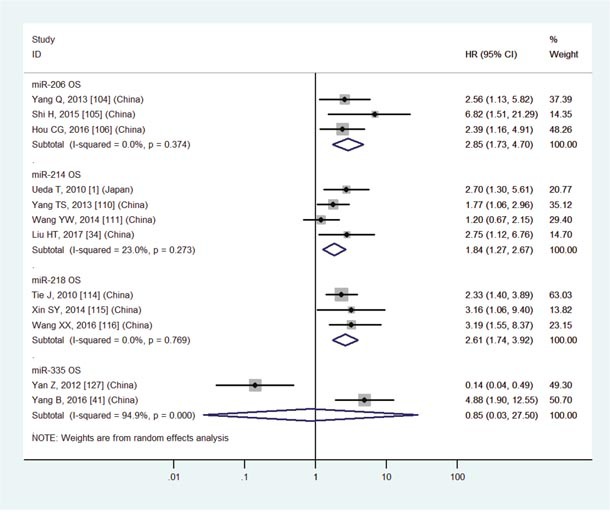
Forest plot of the analyses about high expression of miR-214 or low expression of miR-206, 218, 335 and OS

**Figure 8:**
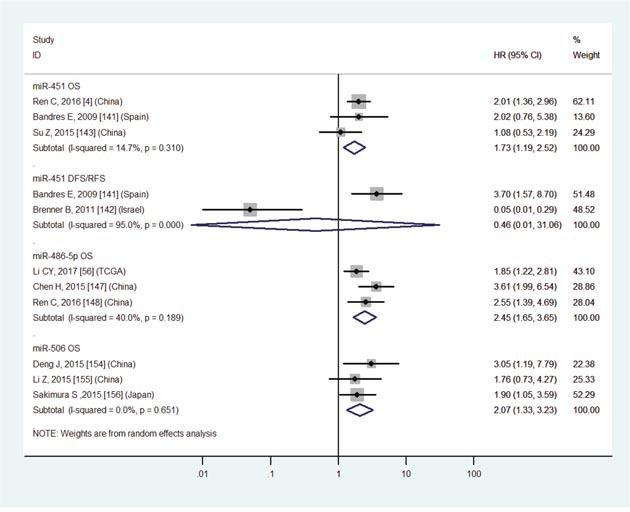
Forest plot of the analyses about low expression of miR-451, 486-5p, 506 and OS or DFS/RFS

The details were shown in Table 3 and Figures 3-8.

### No significant association between high expression of miR-20a, 107, 143, 150, 192 or low expression of miR-27b, 183, 200c, 335 and OS

The details were shown in Table 3 and Figures 3-7.

## DISCUSSION

### Present situation

Increasing evidence has shown that various miRNAs are associated with survival outcome in GC patients [1–167]. However, inconsistent results have emerged. For example, expression levels of miR-200c are up-regulated in blood [98, 99] but down-regulated [66, 96] in tissue compared with normal samples. Furthermore, expression levels of miR-214 [1, 34, 110, 111] and miR-451 [4, 141–143] are unsteadily expressed (up or down). Surprisingly, there are significant associations between aberrant expression levels of them and OS (P<0.05, Table 3, Figures 7 and 8). Therefore, it is essential to conduct a meta-analysis to better understand associations between expression levels of miRNAs and prognosis of GC patients.

### Main findings

We performed the meta-analyses about 26 miRNAs and OS. As the most studied miRNA, GC patients with high miR-21 expression have a significantly poorer OS than those with low miR-21 expression (P<0.05). But there is no significant association between high miR-21 expression and RFS/CSS (P=0.17). According to our reference standard, miR-21 is still considered to be a significantly prognostic biomarker. There are some other miRNAs with significantly prognostic value in GC, including miR-20b, 106b, 125a, 137, 141, 145, 146a, 196a, 196b, 206, 214, 218, 451, 486-5p and 506 (P<0.05). Among them, miR-20b, 125a, 137, 141, 146a, 196a, 206, 218, 486-5p and 506 are strong biomarkers of prognosis in GC (HR≥2).

### Molecular mechanisms for studied miRNAs

In addition to the findings mentioned above, a summary of miRNAs with altered expression, their potential targets and pathways entered this study is detailed in Table 4. It is remarkable that there is functional overlapping or connection among those miRNAs. Twenty miRNAs (miR-20a, 27b, 34a, 106b, 107, 125a, 137, 141, 143, 146a, 183, 192, 196a, 196b, 200c, 214, 218, 335, 451 and 506) are involved in cell functions, including cell apoptosis, colony formation, cycle, differentiation and so on. Zhou et al. [66] reported that miR-200c/141 likely increased E-cadherin expression indirectly through down-regulating ZEB1/2, indicating that this pathway may participate in GC migration and invasion. Additionally, Tsai et al. [91] found that GC cell migration and invasion was enhanced by overexpression of miR-196a/-196b and radixin was recognized as a target of miR-196a/-196b. In a word, these relationships may be involved in the progression of GC.

**Table 4:** Summary of miRNAs with altered expression, their potential targets and pathways entered this study

miRNA	Reference	Expression	Potential target	Pathway
20a	3,5,11	Up	E2F1, HIPK1	Cell differentiation, proliferation, self-renewal and Wnt/β-catenin signaling
20b	3,12,13	Up	None	None
21	3,6,14-19	Up	None	None
27b	32-34	Down	CCNG1, VEGF-C	Cell migration and proliferation
34a	3,38-41	Down	MET, Survivin	Cell apoptosis, colony formation, invasion and proliferation
106b	3,6,45	Up	PTEN	Cell invasion and migration
107	3,46,47	Up	DICER1	Cell invasion and migration
125a	49-51	Down	VEGF-A, ERBB2	Cell proliferation
137	62-64	Down	KLF12, MYO1C, CDK6	Cell cycle, differentiation, migration and proliferation
141	65-67	Down	ZEB1/2, E-cadherin, IGF1R	Cell colony formation, cycle, invasion, migration, viability and TGF-β/ZEB signaling
143	3,69,70	Down	BACH1	Cell invasion, proliferation and TGF-β/Mad signaling
145	34,56,72,73	Down	α-SMA	None
146a	74-76	Down	EGFR, IRAK1, LIN52	Cell apoptosis, invasion, migration and proliferation
150	12,77,79	Up	None	None
183	56,83,84	Down	EZR, BMI1	Cell colony formation, invasion and proliferation
192	48,79,87	Up	None	Cell invasion
196a	88,90-92	Up	CDKN1B, Rdx	Cell colony formation, cycle, invasion, migration and proliferation
196b	56,91-93	Up	Rdx	Cell invasion and migration
200c	98,9966,96	Up (blood)Down (tissue)	ZEB1/2, E-cadherin	Cell invasion, migration and TGF-β/ZEB signaling
206	104-106	Down	None	None
214	1,34,110,111	Up or Down	CSF1, PTEN	Cell invasion, migration and proliferation
218	114-116	Down	ROBO1	Cell invasion and sli/ROBO1 signaling
335	41,127,128	Down	Survivin, BIRC5, CRKL	Cell apoptosis, cycle, growth, invasion, migration and proliferaion
451	4,141-143	Up or Down	MIF	Cell invasion, migration and proliferation
486-5p	56,147-148	Down	FGF9	None
506	154-156	Down	Yap1, ETS1, SNAI2	Cell epithelial-mesenchymal transition, growth, invasion, migration and proliferation

E2F1: E2F transcription factor 1; HIPK1: homeodomain interacting protein kinase 1; CCNG1: cyclin G1; VEGF: vascular endothelial growth factor; PTEN: protein tyrosine phosphatase and tensin homologue; DICER1: dicer 1, ribonuclease type III; ERBB2: erb-b2 receptor tyrosine kinase 2; KLF12: krűppel-likefactor 12; MYO1C: myosin 1C; ZEB1/2: zinc finger E-boxbinding homeobox 1/2; IGF1R: insulin-like growth factor 1 receptor; BACH1: BTB domain and CNC homolog 1; α-SMA: α smooth muscle actin; EGFR: epidermal growth factor receptor; IRAK1: interleukin 1 receptor associated kinase 1; LIN52: lin-52 homolog (C. elegans); EZR: ezrin; BMI1: BMI1 proto-oncogene, polycomb ring finger; CDKN1B: cyclin dependent kinase inhibitor 1B; Rdx: radixin; CSF1: colony stimulating factor 1; Robo1: roundabout guidance receptor 1; BIRC5: baculoviral IAP repeat containing 5; CRKL: CRK like proto-ongogene, adaptor protein; MIF: macrophage migration inhibitory factor (glycosylation-inhibiting factor); FGF9: fibroblast growth factor 9; YAP1: Yes associated protein 1; ETS1: ETS proto-oncogene 1, transcription factor; SNAI2: snail family transcriptional repressor 2; TGF-β: transforming growth factor-β; Mad: mothers against dpp; AKT1: AKT serine/threonine kinase 1; sli: slit.

### Strengths of the meta-analysis

This meta-analysis has several strengths which are as follows: (1) we searched almost all articles with survival outcomes in GC patients with diverse miRNAs. Moreover, the present expression profile of miRNAs was clearly listed in Table 1 in terms of names of miRNAs; (2) articles measuring at least one of survival curves about OS, CSS, DFS, RFS, PFS and MFS were finally included and articles only reporting HR or 95%CI without any of survival curves were excluded by us; (3) miRNAs investigated more than or equal to 3 times were conducted meta-analyses; (4) almost all sample sizes of included studies are more than or equal to 30 (except 1 study [64]), enhancing the power and broadening the applicability of the outcomes to GC patients.

### Limitations

However, one should keep in mind the following limitations: (1) 1 miRNA considered as significant biomarker of prognosis contained a high heterogeneity (miR-21); (2) there are many variables among the present meta-analysis, such as different types of samples (tissue, plasma and serum), disease stages, cut-off values and miRNA methods; (3) our meta-analysis only included English articles, which might exclude certain relevant articles with other languages; (4) articles only reporting HR or 95%CI without survival curves were excluded by us, reducing the sample sizes of included articles; (5) as a result of substantial relevant articles and data about GC, we subjectively and selectively included some researches according to the criteria of inclusion and exclusion (Table 5), leading to ignore a few potential miRNAs with prognostic value.

**Table 5:** Information of search methods and criteria of inclusion and exclusion

Methods	Information
Search strategy	4 search engines, including PubMed, EMBASE, Web ofScience and Cochrane Database of Systematic Reviews
Search deadline	March 19, 2017
Search term	mir and gastric cancer
Inclusion criteria	(1) Patients with gastric cancer;(2) Expression of miRNAs and survival outcome intissue, plasma or serum were measured;(3) At least, one of survival curves about overall survival(OS), cause-specific survival (CSS), disease-free survival(DFS), recurrence-free survival (RFS), progression-freesurvival (PFS) and metastasis-free survival (MFS)was measured, with or without the HR or 95%CI;(4) Full text articles in English
Exclusion criteria	(1) Reviews, letters or laboratory studies withoutoriginal data and retracted articles;(2) Frequency of studies estimating prognostic valueof miRNAs ≤2;(3) Studies which cannot be merged;(4) If more than one article had been published on theidentical study cohort, only the most comprehensivestudy was selected for the present meta-analysis

### Implications for future clinical and scientific research

It is worth mentioning that this meta-analysis is the first systematic estimation of the relevance between miRNA expression and prognosis of GC patients. There are some implications for future clinical and scientific research in the present meta-analysis: (1) for clinical doctors and other healthcare providers, combined detection of miRNA expression can greatly enhance the estimation about survival time of GC patients and timely treatment can be offered; (2) for scientific researchers, the present study trend on associations between miRNAs and prognosis of GC patients can be conveniently seen in Table 1. As a result, selectively basic experiments can be performed by them (Table 4); (3) inconsistent outcomes of prognosis about miRNAs may be solved according to the basement of the current meta-analysis.

## MATERIALS AND METHODS

### Search strategy, inclusion criteria and exclusion criteria

The details were presented in Table 5. Two authors (Yue Zhang and Dong-Hui Guan) independently performed this comprehensive online search.

### Quality assessment

Yue Zhang and Dong-Hui Guan confirmed all eligible investigations that analyzed the prognostic value of miRNAs in GC, and Yue-Hua Jiang reassessed uncertain data.

### Statistical analysis

All analyses were conducted using Stata version 13.0 (StataCorp, College Station, Texas, USA). The relative effect sizes for HR were characterized as moderate (protective [0.51-0.75] or contributory [1.35-1.99]) and large (≤0.50 or≥2). The HR was considered significant at the P<0.05 level if the 95%CI did not include the value 1. If the P values from OS and other survival results about corresponding miRNAs were inconsistent, the HR from OS was considered to the main reference standard. Because different types of samples (tissue, plasma and serum) from GC patients at different disease stages, cut-off values and miRNA methods were used in individual studies, random-effects models (DerSimonian-Laird method) were more appropriate than fixed-models (Mantel-Haenszel method) for most of the analyses. Consequently, the random-effects models were used in the current meta-analysis. Publication bias was estimated using the Begg's funnel plot. A two-tailed P value <0.05 was considered significant. Sensitivity analysis (influence analysis) was carried out to test how powerful the combined effect size was to removal of individual investigations. If the point assessment was out the 95%CI of the pooled effect size after it was removed from the analysis, an individual study was doubted to have excessive influence.

## CONCLUSIONS

In summary, miR-20b, 21, 106b, 125a, 137, 141, 145, 146a, 196a, 196b, 206, 214, 218, 451, 486-5p and 506 demonstrate significantly prognostic value. Among them, miR-20b, 125a, 137, 141, 146a, 196a, 206, 218, 486-5p and 506 are strong biomarkers of prognosis in GC.

Acquisition of data: Yue Zhang and Dong-Hui Guan.

Analysis and interpretation of data: Yue Zhang, Dong-Hui Guan, Rong-Xiu Bi and Jin Xie.

Drafting of the manuscript: Yue Zhang.

Revision of manuscript: Yue Zhang, Dong-Hui Guan, Rong-Xiu Bi, Jin Xie, Chuan-Hua Yang and Yue-Hua Jiang.

Supervision of work: Rong-Xiu Bi, Jin Xie, Chuan-Hua Yang and Yue-Hua Jiang.

All authors read and approved the final manuscript.

Role of funding source: The funder had no role in study design, data collection and analysis, decision to publish, or preparation of the manuscript.
